# A Model for Monitoring Spontaneously Reported Medication Errors Using the Adjuvanted Recombinant Zoster Vaccine as an Example

**DOI:** 10.1155/2024/6435993

**Published:** 2024-01-24

**Authors:** Christophe Dessart, Fernanda Tavares-Da-Silva, Lionel Van Holle, Olivia Mahaux, Jens-Ulrich Stegmann

**Affiliations:** ^1^Global Safety, GSK, Wavre 1300, Belgium; ^2^OpenSourcePV, Court-Saint-Etienne 1490, Belgium

## Abstract

A European legislation was put in place for the reporting of medication errors, and guidelines were drafted to help stakeholders in the reporting, evaluation, and, ultimately, minimization of these errors. As part of pharmacovigilance reporting, a proper classification of medication errors is needed. However, this process can be tedious, time-consuming, and resource-intensive. To fulfill this obligation regarding medication errors, we developed an algorithm that classifies the reported errors in an automated way into four categories: potential medication errors, intercepted medication errors, medication errors without harm (i.e., not associated with adverse reaction(s)), and medication errors with harm (i.e., associated with adverse reaction(s)). A fifth category (“conflicting category”) was created for reported cases that could not be unambiguously classified as either potential or intercepted medication errors. Our algorithm defines medication error categories based on internationally accepted terminology using the Medical Dictionary for Regulatory Activities (MedDRA®) preferred terms. We present the algorithm and the strengths of this automated way of reporting medication errors. We also give examples of visualizations using spontaneously reported vaccination error data associated with the adjuvanted recombinant zoster vaccine. For this purpose, we used a customized web-based platform that uses visualizations to support safety signal detection. The use of the algorithm facilitates and ensures a consistent way of categorizing medication errors with MedDRA® terms, thereby saving time and resources and avoiding the risk of potential mistakes versus manual classification. This allows further assessment and potential prevention of medication errors. In addition, the algorithm is easy to implement and can be used to categorize medication errors from different databases.

## 1. Introduction

Medication errors are defined as unintended failures in the drug treatment or vaccination process (during prescription, storage, distribution, preparation, or administration) that lead to, or have the potential to lead to, harm to the patient [1]. As it has been estimated that about 18.7%–56.0% of adverse events (AEs) among hospitalized patients are a result of preventable medication errors, they have been recognized as a major public health burden [1]. These errors can occur with any medicinal product, at any step in patient care, and in any care setting [2]. The most common medication errors in hospitals are prescription and administration errors, which account for about 75% of medication errors in this setting [2]. Some examples include prescription for or administration to the wrong patient, failure to prescribe an indicated medication or prescribing without indication, administration of an inappropriate dose or via the wrong route, and failure to administer the medication when due [2, 3]. Several factors can influence the incidence of medication errors. These factors can be related to the medication itself (e.g., similar sounding names or a low therapeutic index), to the patient (e.g., age, comorbidities, nonadherence to the medication, impaired cognition, or polypharmacy), or to the healthcare professional (e.g., use of abbreviations in prescriptions, unclear handwriting, or lack of up-to-date knowledge) [2, 3].

To prevent medication errors, a process of care should be designed to ensure patients are protected against these errors and their potential harm. This can be achieved by ensuring efforts are made by regulatory authorities and manufacturers (e.g., clear product label information), by ensuring an up-to-date medication list, by developing educational programs or by using information technology (e.g., drug databases or computerized physician order entry) [3]. It is important to monitor and analyze medication errors, whether they result in harm or not. Findings must be communicated to improve the process of care and ensure that the risk of such errors can be minimized throughout the product life cycle [2, 4]. Since 2012, the European Union pharmacovigilance legislation requires the reporting of all AEs resulting from medication errors to EudraVigilance [5]. To support stakeholders involved in the reporting, evaluation, and prevention of medication errors with implementing this legislation, the European Medicines Agency (EMA) published a good practice guide in 2015 to improve recording, coding, reporting, and assessment of medication errors, regardless of their association with AEs. Intentional overdose, off-label use, misuse, and abuse are not in the scope of this guide [1].

As part of pharmacovigilance reporting, a proper classification of medication errors is needed [1]. This process can be tedious, time-consuming, and resource-intensive. To facilitate this classification, GlaxoSmithKline (GSK) has developed an algorithm that allows automatic categorization of medication errors from its spontaneous report database, based on Medical Dictionary for Regulatory Activities (MedDRA®) preferred terms (PTs).

The current paper describes the algorithm and its advantages and limitations. It also gives examples of possible visualizations of medication errors using a previously described tool [6]. As an example, we present vaccination errors associated with the adjuvanted recombinant zoster vaccine (RZV, *Shingrix*, GSK), which was first licensed in October 2017 [7–9]. This vaccine was chosen as prior experiences with other vaccines indicated that reports of vaccination errors are highest in the period shortly after licensure [10]. RZV entered the market when another vaccine (*Zostavax*, Merck Sharp, and Dome) requiring different storage conditions, preparation, and administration procedures had already been available for a decade [11, 12]. We previously observed that the lack of familiarity with the RZV vaccine likely contributed to vaccination errors [13]. The use of the visualizations helped us to quickly identify and gain insights into the types of errors reported with this vaccine and to identify potential areas where preventive measures were beneficial [13].

## 2. Materials and Methods

GSK collects spontaneous reports of all AEs following immunization with its vaccines in its worldwide safety database as per good pharmacovigilance practices. These spontaneous report data from unsolicited communications describing one or more AEs in vaccinated patients are either submitted to GSK directly and voluntarily by individual reporters (e.g., healthcare professionals, regulatory authorities, or consumers, who report for themselves or others) or are identified in the scientific literature or interactive digital media as single case reports [6]. AEs and medication errors are encoded in the database using the MedDRA® terminology, the international medical terminology developed under the auspices of the International Council for Harmonisation of Technical Requirements for Pharmaceuticals for Human Use (ICH), in line with EMA's good practice guide [1].

Medication errors may trigger a series of events, and more than one stage in the treatment process may be affected by an error. For example, a prescription error can lead to a dispensing error and consequently result in an administration error. Therefore, one spontaneous report (further referred to as a “case”) can contain more than one medication error term in MedDRA®. It is important to capture the primary error and any subsequent errors reaching the patient and to assess the clinical consequences for the patient. Likewise, for a given medication (e.g., a vaccine), more than one dose can be recorded in a case, with medication errors reported after each dose. This algorithm has the advantage of classifying the same case into different categories at the product and dose level. Hence, if more than one product is recorded as suspect (e.g., in the case of vaccine co-administration), the algorithm will distinguish which product has been administered incorrectly.

**Figure 1:**
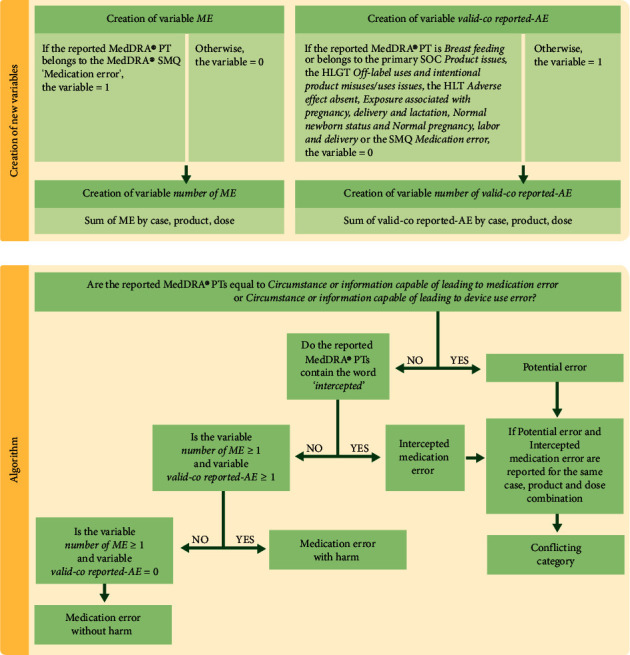
Flowchart for the creation of new variables and the algorithm. AE, adverse event; HLGT, high level group term; HLT, high-level term; ME, medication error; MedDRA®, medical dictionary for regulatory activities; PT, preferred term; SMQ, standardized MedDRA® query; and SOC, system organ class.

For a reported case, each dose of a selected product is classified by the algorithm into one of the four main categories of medication errors, in line with the EMA's good practice guide [1]: potential medication errors, intercepted medication errors, medication errors without harm (i.e., not associated with adverse reaction(s)) and medication errors with harm (i.e., associated with adverse reaction(s)) (Table 1). A fifth category (“conflicting category”) was created for reported cases that could not be unambiguously classified as either potential or intercepted medication errors (Table 1). For these cases, corrections to the database entry should be requested to the case management group. A flowchart is presented in Figure 1, and the code can be found in the S1 Supplementary Materials.

Events coreported with a medication error are by default considered harm, regardless of whether they are caused by the medication error, except for the following MedDRA® PTs (i.e., nonvalid-coreported AE): all MedDRA® PTs belonging to the primary system organ class “*product issues*,” the high level group term “*off-label uses and intentional product misuses/uses issues*,” the high level terms (HLT) “*adverse effect absent*,” “*exposure associated with pregnancy, delivery and lactation*,” “*normal newborn status*” and “*normal pregnancy, labor and delivery,*” or the PT “*breastfeeding*,” which are not adverse reactions/AEs. The selection of the MedDRA® PTs can be adapted according to MedDRA® version updates.

The automated process allows categorization of new cases as well as retrospective categorization in searches. To validate the algorithm, two methods for categorizing medication errors were compared, one using the algorithm and the other by screening the cases manually. While we found comparable results for categorization with both methods, manual screening was shown to be time-consuming and subject to human errors.

## 3. Results

### 3.1. Visualization of Medication Errors

Regular and standardized review of safety data is essential. Therefore, GSK routinely performs quantitative signal detection for its products. A quantitative signal of disproportionate reporting at the MedDRA® PT level for a vaccine-event pair is generated if the lower limit of the 95% confidence interval of the stratified (by sex, age group, geography, and reporting period) proportional reporting ratio is above 2, and at least 3 cases are reported. These quantitative signals, along with relevant visualizations, are made available on a previously described customized web-based platform, the signal mining and management (SMM) tool [7]. Different algorithms and visualizations are integrated into this tool to ease medical review and data analysis, which includes mining the raw data and signals, looking at trends, testing hypotheses, reviewing clinical details of cases, etc. Visualizations specific to medication errors have been developed in the SMM tool, as further elaborated below.

To illustrate the strengths and possibilities of developing visualizations on top of the automatic categorization of medication errors by our algorithm, we present and describe the visualizations for the category “medication error without harm,” using spontaneous reports associated with RZV as an example. We do not aim to discuss specific findings here but merely want to use this example to illustrate the methodology.

**Figure 2:**
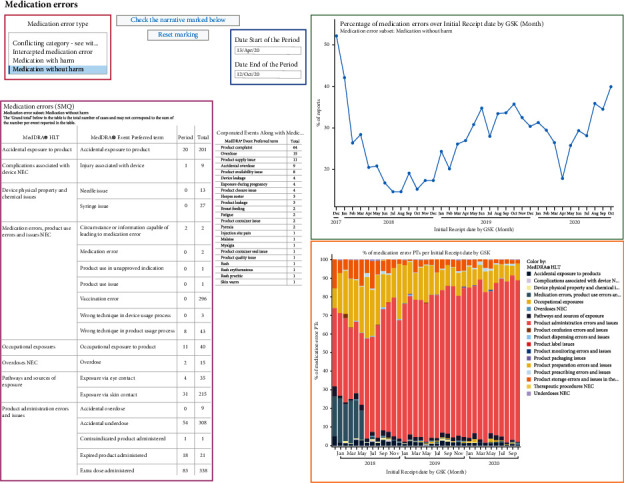
Example of visualizations in the SMM tool: overall view. HLT, high-level term; MedDRA®, medical dictionary for regulatory activities; NEC, not elsewhere classified; PT, preferred term; SMM, signal mining and management; and SMQ, standardized MedDRA® query.

**Figure 3:**
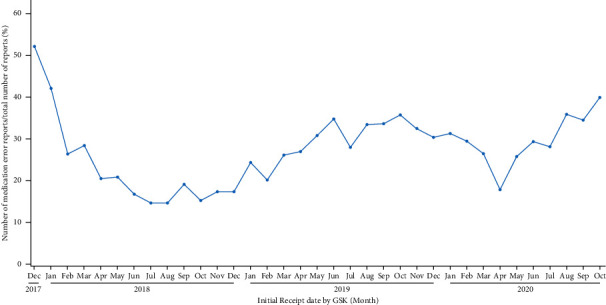
Evolution of the ratio of medication errors without harm over all spontaneous reports for the selected vaccine.

**Figure 4:**
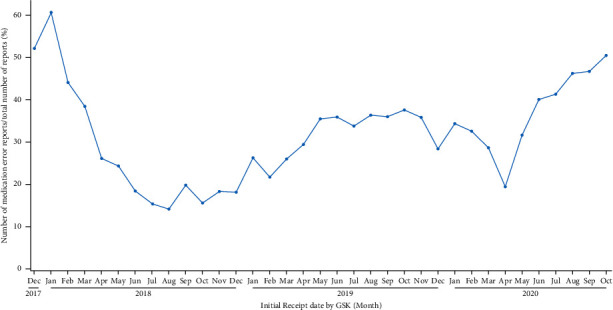
Evolution of the ratio of medication errors without harm over all spontaneous reports for the selected vaccine in the United States.

Figure 2 shows an overall view of the different visualizations as they appear in the SMM tool. Data are shown for a specific period of time (“period”), which can be selected in the tool (Figure 2, blue box) and can be compared to the cumulative period (“total,” from entry of the first case in the database until the date of freezing). A drop-down menu allows the user to visualize the information for one of the predefined medication error categories (Figure 2, red box). This information includes a tabular view of medication errors by MedDRA® PT (grouped by MedDRA® HLT) (Figure 2, purple box) and graphs depicting the evolution over time of reported medication errors (Figure 2, green and orange boxes, Figures 3 and 4). To ease the data review and its documentation, the table with medication errors by PT (Table 2) can be exported in a different format (i.e., pdf).

Visualization of the evolution over time of the ratio of medication errors without harm over all spontaneous reports for the selected vaccine in the database (Figure 2, green boxes, Figure 3) allows the identification of trends in support of safety signal detection. Proportions rather than absolute numbers are shown because changes in absolute numbers can be the result of varying reporting habits or variations in the number of administered doses. Such changes are expected to have an equivalent impact on the number of medication errors and on the number of all other spontaneous reports and would therefore not have a major impact on their ratio. The graphical representation shown in Figure 3 enabled the immediate identification of a safety signal due to the high proportion of medication error reports shortly after the initial launch of RZV (52% of all spontaneous reports associated with RZV worldwide). The safety signal was mainly a result of an incorrect route of administration, the wrong reconstitution of the vaccine, or the wrong storage conditions [13]. The identification of this signal triggered the introduction of corrective actions (e.g., implementation of educational programs and product label information clarification), which led to a decrease in the proportion of medication errors [6, 13]. To better understand these data and possible safety signals, the data can also be visualized at the country level (Figure 4).

**Figure 5:**
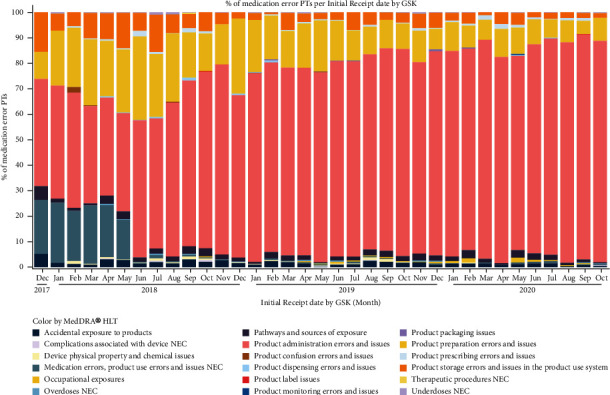
Evolution of the type of medication errors (as a percentage of all reported medication errors) over time. MedDRA® HLT, medical dictionary for regulatory activities high-level term; NEC, not elsewhere classified; and PT, preferred term.

**Figure 6:**
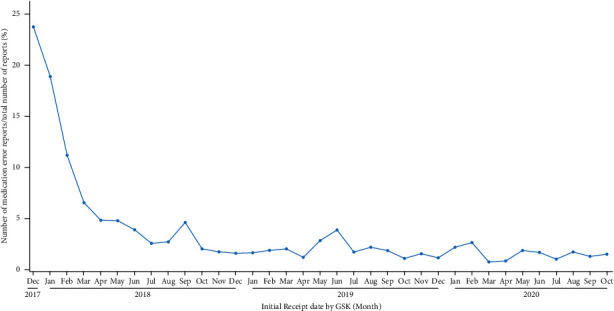
Evolution of the ratio of case reports with an incorrect route of administration over all reported cases.

**Figure 7:**
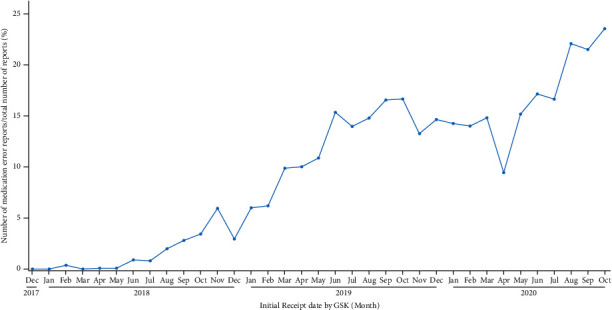
Evolution of the ratio of case reports with an incomplete schedule over all reported cases.

Such visualization, as shown in Figure 3, also allowed the detection of an increase in the reporting of medication errors on two other occasions: the first one starting in January 2019 and the second one starting more recently, in May 2020. To identify the specific type of medication error responsible for the observed increases in reports, we looked at the visualization of the different types of medication errors by MedDRA® HLT as a percentage of all medication errors over time (Figure 2, orange box, Figure 5). We saw that the most frequently reported error was mainly linked to product administration errors and issues. Further analyses allowed us to determine that while the reporting of events such as “incorrect route of administration” remained stable (Figure 6), an increase was seen for the MedDRA® PTs “incomplete course of vaccination” or “inappropriate schedule of product administration” (Figure 7). This increase was assumed to result from the product supply issue GSK faced in 2019 and, more recently, from the restrictions imposed by the coronavirus disease 2019 (COVID-19) pandemic that prevented individuals from receiving their second dose.

For the category “medication error with harm,” the most frequently coreported AEs can also be visualized (Table 3). A comparison can then be made between the safety profile linked to the medication error and the known safety profile of the product when administered according to the label.

## 4. Discussion

The broadening of the definition of a signal to include medication errors reflects major efforts to reduce the burden of harm from medication errors and protect patient safety. As part of our efforts to fulfill our obligation under the legislation and good practice guide, an algorithm that can be used to categorize medication errors reported to different databases was developed. To our knowledge, such an algorithm to categorize medication errors was not available.

The algorithm allows in-stream and retrospective categorization of medication errors via an automated process, minimizing the risk of mistakes. It has already proven its use as it enabled the identification of a safety signal related to medication errors after the launch of RZV, thereby allowing the implementation of measures to minimize the risk of medication errors [6, 13]. The algorithm could be useful in the context of the current COVID-19 pandemic, as several vaccines with different storage requirements, preparation schedules, and administration schedules are on the market [14]. Moreover, while the algorithm is currently being used in the context of vaccines, we strongly believe that its usefulness can also be extended to other medicinal products due to its straightforward implementation. All medication error data are routinely discussed in periodic safety update reports due to regulatory requirements.

Several benefits are associated with the algorithm. The manual review of medication errors is time-consuming, requires human resources, and is prone to errors, and coding conventions may change over time. The implementation of this algorithm may help circumvent these issues as it categorizes cases in an automated way. Additionally, it only requires some adaptations that can easily be implemented when there are changes in legislation (e.g., requiring other information on medication errors to be collected, reported, and analyzed) or updates to any MedDRA® version. As the algorithm is based on internationally accepted terminology using MedDRA®, the categorization is always done in a consistent way. This increases the quality of categorization by decreasing the subjectivity of reviewing and categorizing medication errors by different reviewers. This automated process can also minimize the risk of mistakes with manual classifications. The algorithm allows a continuous categorization of new and existing cases entered in the database, as new information arises. With the implementation of the new ICH-E2B (R3) reporting format in the EudraVigilance system in November 2017 [15], it is also possible to identify which of the reported suspect medications was actually involved in the error, although this type of information is not always available. Before the implementation of the European legislation, the categorization of case reports required a manual review of the cases with no re-evaluation after processing. By implementing this algorithm, the categorization of medication errors is performed in the same way for all case reports. In addition, data integrity issues can be flagged automatically. For example, when intercepted error and potential error are coded for the same case, the case will be categorized in a separate “conflicting category,” which will trigger further follow-up and corrections in the database. Finally, the use of predefined visualizations allows for quick identification of signals and monitoring of changes over time.

The limitations of the algorithm are mainly linked to coding issues or maintenance of the list of terms. Indeed, the algorithm is based on coding practices, which can contain errors and inconsistencies, while the implementation of the algorithm requires correct and consistent coding. Hence, it is important to have rules in place to flag conflicting coding. Moreover, the list of nonvalid-coreported-AEs requires maintenance following any MedDRA® version update to ensure they are still accurate, although this could be tackled by the creation of a Standardized MedDRA® Query. Due to the nature of spontaneous reports, the evaluation of medication errors can be used to quickly identify and gain insights into the types of errors reported and to identify potential areas where preventive measures could be beneficial, rather than to quantify the risks associated with medication errors. Another limitation with the use of this algorithm is that no causality assessment is performed. Therefore, when multiple medications are involved as suspects in one report of medication error with harm, it is not always possible to identify which of the reported suspect medications was involved in the error. Finally, as no other methods/algorithms are freely available, this algorithm was only compared with the manual method.

## 5. Conclusions

A new algorithm to categorize medication errors in an automated way was developed. This algorithm can be applied to different databases as it is easy to implement and is thought to facilitate the assessment of medication errors. In addition, it has already proven its use, as it enabled the identification of a safety signal related to medication errors after the launch of RZV.

## Figures and Tables

**Table 1:** General principles of the algorithm used to categorize medication errors for a dose of product X (MedDRA® version 23.1).

Category	Algorithm
Potential errors	If one of the reported MedDRA® PTs is “*Circumstance or information capable of leading to medication error*,” the case is categorized as potential error for the dose of product X
Intercepted medication errors	If one of the reported MedDRA® PTs contains the word “*intercepted”* and the MedDRA® PT “*Circumstance or information capable of leading to medication error*” was not reported after a dose of product X, the case is categorized as intercepted medication error for the dose of product X
Medication errors without harm	If there is no potential error or intercepted medication error and all MedDRA® PTs reported belong to the SMQ “*medication error*,” the case is categorized as medication error without harm for the dose of product X
Medication errors with harm	If there is no potential error or intercepted medication error, at least one valid event belonging to the SMQ “*medication error*” and at least one event not belonging to the SMQ “*medication error*,” the case is categorized as medication error with harm for the dose of product X
	The following MedDRA® PTs are not considered valid events to be used for the definition of medication error with harm: the MedDRA® PTs belonging to the primary SOC “*product issues*,” the HLGT “*off-label uses and intentional product misuses/uses issues*,” the HLTs “*adverse effect absent*,” “*exposure associated with pregnancy, delivery and lactation*,” “*normal newborn status*” and “*normal pregnancy, labor and delivery*” or the PT “*breastfeeding*”
Conflicting category	For a dose of product X, if one of the reported MedDRA® PTs contains the word “*intercepted”* and the PT “*circumstance or information capable of leading to medication error*,” the case is categorized under conflicting category and should be corrected

HLGT, high-level group term; HLT, high-level term; MedDRA®, medical dictionary for regulatory activities; PT, preferred term; SMQ, standardized MedDRA® query; SOC, system organ class.

**Table 2:** Example of table with reported medication errors by MedDRA® HLT and PT for the category medication without harm.

MedDRA® HLT	MedDRA® PT	Period^a^	Total^b^
Accidental exposure to product	Accidental exposure to product	20	201

Complications associated with device NEC	Injury associated with device	1	9

Device physical property and chemical issues	Needle issue	0	13
	Syringe issue	0	27

Medication errors, product use errors and issues NEC	Circumstance or information capable of leading to medication error	2	2
	Medication error	0	2
	Product use in unapproved indication	0	1
	Product use issue	0	1
	Vaccination error	0	296
	Wrong technique in device usage process	0	3
	Wrong technique in product usage process	8	43

Occupational exposures	Occupational exposure to product	11	40

Overdoses NEC	Overdose	2	15

Pathways and sources of exposure	Exposure via eye contact	4	35
	Exposure via skin contact	31	215

Product administration errors and issues	Accidental overdose	0	9
	Accidental underdose	54	308
	Contraindicated product administered	1	1
	Expired product administered	18	21
	Extra dose administered	83	338
	Inappropriate schedule of product administration	376	2016
	Incomplete course of vaccination	1290	4025
	Incorrect dose administered	199	1951
	Incorrect route of product administration	49	675
	Product administered at inappropriate site	8	49
	Product administered to patient of inappropriate age	41	218
	Product administration error	5	17
	Wrong product administered	10	68

Product confusion errors and issues	Product label confusion	0	4
	Product name confusion	0	1
	Product packaging confusion	0	4

Product dispensing errors and issues	Product dispensing error	2	14

Product label issues	Product label issue	1	2

Product monitoring errors and issues	Labelled drug-disease interaction medication error	1	1

Product packaging issues	Product packaging issue	0	6

Product preparation errors and issues	Product preparation error	37	737
	Product preparation issue	161	1283

Product prescribing errors and issues	Contraindicated product prescribed	1	1
	Product prescribing error	18	76

Product storage errors and issues in the product use system	Product storage error	65	680

Therapeutic procedures NEC	Interchange of vaccine products	0	1

Underdoses NEC	Underdose	5	39

Grand total^c^		2189	10504

HLT, high-level term; MedDRA®, medical dictionary for regulatory activities; NEC, not elsewhere classified; PT, preferred term. ^a^Data for a chosen period. ^b^Data from entry of the first case in the database until date of database freezing. ^c^Total number of distinct cases; may not correspond to the sum per event reported in the table as different MedDRA® PTs may be reported in the same case.

**Table 3:** Example of table with most frequent coreported adverse events for the category medication errors with harm.

MedDRA® PT	Total
Injection site pain	264
Injection site erythema	233
Pain in extremity	170
Pyrexia	154
Pain	151
Injection site swelling	148
Headache	124
Chills	101
Erythema	101
Herpes zoster	101
Fatigue	99
Influenza-like illness	76
Rash	67
Injection site warmth	62
Myalgia	60
Arthralgia	57
Pruritus	57
Nausea	53
Malaise	48
Peripheral swelling	46
Vaccination failure	42
Swelling	41
Injected limb mobility decreased	33
Injection site pruritus	24
Neuralgia	24
Injection site rash	23
Urticaria	23
Extensive swelling of vaccinated limb	22
Paresthesia	22
Insomnia	20
Overdose	20
Asthenia	18
Dizziness	17
Feeling hot	17
Injection site reaction	17
Skin warm	17
Blister	15
Contusion	15
Diarrhea	15
Injection site bruising	15
Injection site induration	15
Rash vesicular	15
Feeling abnormal	14
Injection site hemorrhage	14
Hypoesthesia	13
Neck pain	13
Back pain	12
Rash erythematous	12
Somnolence	12
Hyperhidrosis	11
Local reaction	11
Condition aggravated	10
Illness	10
Injection site cellulitis	10
Injection site discomfort	10
Tenderness	10
Vomiting	10

MedDRA®, medical dictionary for regulatory activities; PT, preferred term.

## Data Availability

The data that supports the findings of this study are available in the supplementary materials of this article.

## Additional Points

*Plain Language Summary*. The success of any drug treatment depends in part on the medication being handled and administered correctly. Errors can occur at any step of the process: incorrect medication, dose, schedule, or route of administration; improper storage or preparation of the medication; or prescription for or administration to the wrong patient. These unintentional mishaps, called medication errors, can potentially be harmful to patients. Monitoring medication errors can be key to reducing their occurrence and consequences. We therefore developed an algorithm to classify medication errors. It allows the automatic classification of medication errors into different categories in a standardized manner. Our algorithm is easy to implement and allows rapid and consistent categorization of medication errors, thereby saving time and human resources and improving the assessment of medication errors. *Trademarks*. *Shingrix* is a trademark owned by or licensed to the GSK group of companies. *Zostavax* is a trademark of Merck Sharp and Dohme. The MedDRA® trademark is registered by ICH.

## Supplementary Materials

Supplementary Materials Algorithm code.
