# Supplementary material for: A Model for Monitoring Spontaneously Reported Medication Errors Using the Adjuvanted Recombinant Zoster Vaccine as an Example
Source: Adv Pharmacol Pharm Sci. 2024 Jan 24;2024:6435993. doi: 10.1155/2024/6435993 (PMC10830180; doi:10.1155/2024/6435993)
Supplement: Supplementary Materials — Algorithm code. [file 6435993.f1.docx]

**S1 Supplementary Materials 1**

**Algorithm code**

********************************************************************************************************;

* Macro name : MedErr *;

* Version : 1.00 *;

* --------------------------------------------------------------------------------------------------- *;

* Revisions : *;

* Versions Date Author *;

* 1.00 DEC2017 P. Schrauben *;

* Creation of the macro *;

* --------------------------------------------------------------------------------------------------- *;

* Description : *;

* Medication Error coding *;

* --------------------------------------------------------------------------------------------------- *;

* INPUT macro parameters : *;

* inpsrc : name of the case-vaccine-dose-event input dataset (Mandatory) *;

* smqsrc : name of the SMQ input dataset (Mandatory) *;

* mederr_value : value of smq_name that corresponds to medication errors. *;

* Default='MEDICATION ERRORS (SMQ)' *;

* outdsn : name of the output dataset if different from [inpsrc]. (Optionnal) *;

* INPUT datasets : *;

* [inpsrc] : contains case-vaccine-dose-event information. Should at least contain variables: *;

* csc, vcdoseid,vcfamname, evfdot, evsoc,evpt,evhlgt,evhlt *;

* [smqsrc] : contains SMQ information. Restriction to upper case of smq_name=MEDICATION ERRORS (SMQ)*;

* should contain at least evpt and smq_name variables *;

* --------------------------------------------------------------------------------------------------- *;

* OUTPUT dataset : *;

* [inpsrc] : with the medication Error coding variable *;

* OR *;

* [outdsn]: contains the coding variable for medication errors for each case-vaccine-dose *;

* --------------------------------------------------------------------------------------------------- *;

* Variables definition : *;

* csc : Argus case id *;

* vcdoseid : Derived dose number *;

* vcfamname : Product family name *;

* evfdot : Time from first dose to event (TXT) *;

* evsoc : Primary MedDRA system organ class *;

* evpt : MedDRA event preferred term *;

* evhlgt : MedDRA high level group term *;

* evhlt : MedDRA high level term *;

* smq_name : Standard MedDRA query name *;

********************************************************************************************************;

%macro MedErr ( inpsrc = ,

smqsrc = , mederr_value = 'MEDICATION ERRORS (SMQ)',

outdsn =

);

%local i nobs;

%* -------- Checks on macro parameters ---------;

%* ---------------------------------------------;

%* input datasets are filled in and exist?;

%if "&inpsrc." eq "%str()" %then %do;

%put %str(ER)ROR:%str(US)ER: macro &sysmacroname. aborted as macro parameter 'inpsrc' is not filled in, please give the name of the case-vaccine-event input dataset to macro parameter inpsrc=.;

%goto exit;

%end;

%if %sysfunc(exist(&inpsrc.)) eq 0 %then %do;

%put %str(ER)ROR:%str(US)ER: macro &sysmacroname. aborted as [&inpsrc.] dataset does not exist, please check macro parameter inpsrc=.;

%goto exit;

%end;

%if "&smqsrc." eq "%str()" %then %do;

%put %str(ER)ROR:%str(US)ER: macro &sysmacroname. aborted as macro parameter 'smqsrc' is not filled in, please give the name of the SMQ input dataset to macro parameter smqsrc=.;

%goto exit;

%end;

%if %sysfunc(exist(&smqsrc.)) eq 0 %then %do;

%put %str(ER)ROR:%str(US)ER: macro &sysmacroname. aborted as [&smqsrc.] dataset does not exist, please check macro parameter smqsrc=.;

%goto exit;

%end;

%* check if needed variables exists in [smqsrc];

proc contents data=&smqsrc. noprint

out=_cont_smq ( keep = name type format

where = ( lowcase(name) in ("evpt" "smq_name") ) );

run;

%let dsid = %sysfunc(open(_cont_smq));

%let nobs = %sysfunc(attrn(&dsid.,nobs));

%let rc = %sysfunc(close(&dsid.));

%if &nobs. ne 2 %then %do;

%put %str(ER)ROR:%str(US)ER: macro &sysmacroname. aborted as variable evpt or smq_name does not exist in [&smqsrc.], please check input dataset smqsrc=&smqsrc..;

%goto exit;

%end;

%* check if needed variables exists in [inpsrc];

proc contents data=&inpsrc. noprint

out=_cont_inp ( keep = name type format

where = ( lowcase(name) in ("csc" "vcdoseid" "vcfamname" "evfdot" "evsoc" "evpt" "evhlgt" "evhlt") ) );

run;

%let dsid = %sysfunc(open(_cont_inp));

%let nobs = %sysfunc(attrn(&dsid.,nobs));

%let rc = %sysfunc(close(&dsid.));

%if &nobs. ne 8 %then %do;

%put %str(ER)ROR:%str(US)ER: macro &sysmacroname. aborted as needed variable(s) does not exist in [&inpsrc.]:;

%put %str(ER)ROR:%str(US)ER: please check input dataset inpsrc=&inpsrc. that should contain at least csc, vcdoseid, vcfamname, evfdot, evsoc, evpt, evhlgt, evhlt.;

%goto exit;

%end;

%* -------- SMQ Medications errors ---------;

%* -----------------------------------------;

proc sql;

create table _smqMedErr as

select distinct evpt

from &smqsrc.

where upcase(smq_name) eq upcase(&mederr_value.)

order by evpt;

quit;

%* -------- Creation of output dataset ---------;

%* ---------------------------------------------;

/*As per GSK coding convention, the time to onset is put to Not applicable when the event is not relevant for one of the products (e.g., an injection site reaction with an oral vaccine [administered orally] or an event occuring before the exposure to the product). As a result, the condition upcase(evfdot) = 'NOT APPLICABLE' was added to exclude invalid events

The MedDRA event PT ‘Breast feeding’ like the MedDRA SOC 'Product issues', the MedDRA event HLGT 'Off label uses and intentional product misuses/use issues' and the MedDRA HLTs 'Adverse effect absent', 'Exposures associated with pregnancy, delivery and lactation', 'Normal newborn status' and 'Normal pregnancy, labour and delivery' are not to be considered valid adverse events associated to the medication error for the category 'Medication errors with harm'. That PT could have been part of the list but was added afterwards so was added differently however this doesn't impact the output.*/

proc sql;

create table medErr_indiv as

select distinct a.csc, a.vcdoseid, a.vcfamname, a.evsoc, a.evpt, a.evhlgt, a.evhlt, a.evfdot

, case when upcase(evfdot)='NOT APPLICABLE' or upcase(a.evpt)='BREAST FEEDING' then .

when b.evpt ne "" then 1

else 0

end as smq_indiv format=3. length=3

, case when upcase(evfdot)='NOT APPLICABLE' or upcase(a.evpt)='BREAST FEEDING' then .

when b.evpt="" and (evsoc ne 'Product issues' and evhlgt ne 'Off label uses and intentional product misuses/use issues'

and evhlt not in ('Adverse effect absent', 'Exposures associated with pregnancy, delivery and lactation',

'Normal newborn status', 'Normal pregnancy, labour and delivery') ) then 1

else 0

end as nosmqnotin_indiv format=3. length=3

from &inpsrc. as a left join _smqMedErr as b on a.evpt=b.evpt

order by csc, vcdoseid, vcfamname, evsoc, evpt, evhlgt, evhlt;

quit;

proc sql undo_policy=none;

create table medErr_indiv as

select distinct csc, vcdoseid, vcfamname, sum(smq_indiv) as n_validsmq format=3. length=3 label="Nb valid SMQ MedErr"

, sum(nosmqnotin_indiv) as n_nosmqnotin format=3. length=3 label="Nb valid noSMQ MedErr notin"

, evsoc, evpt, evhlgt, evhlt, evfdot, smq_indiv

from medErr_indiv

group by csc, vcdoseid

order by csc, vcdoseid, vcfamname, evsoc, evpt, evhlgt, evhlt, evfdot;

quit;

data medErr_indiv;

set medErr_indiv;

attrib mederr_indiv format=$40.;

if smq_indiv ne . then do;

if evhlgt='Off label uses and intentional product misuses/use issues' then mederr_indiv="OFF";

else if evpt in ('Circumstance or information capable of leading to medication error',

'Circumstance or information capable of leading to device use error') then mederr_indiv='Potential error';

else if index(lowcase(evpt),'intercepted') gt 0 then mederr_indiv='Intercepted medication error';

else if n_validsmq ge 1 and n_nosmqnotin ge 1 then mederr_indiv='Medication with harm';

else if n_validsmq ge 1 then mederr_indiv='Medication without harm';

end;

run;

data %if "&outdsn." ne "%str()" %then &outdsn.;

%else medErr_aggregate; (keep=csc vcdoseid vcfamname n_validsmq n_nosmqnotin mederr);

set medErr_indiv;

by csc vcdoseid;

attrib mederr format=$35. label='Medication error category';

retain mederr;

if first.vcdoseid then mederr=mederr_indiv;

else do;

if mederr="OFF" or mederr_indiv="OFF" then mederr = "OFF";

else if mederr='Conflicting category - see with CMG' then mederr = 'Conflicting category - see with CMG';

else if mederr='Potential error' and mederr_indiv='Intercepted medication error' then mederr = 'Conflicting category - see with CMG';

else if mederr_indiv='Potential error' and mederr='Intercepted medication error' then mederr = 'Conflicting category - see with CMG';

else if mederr='Potential error' or mederr_indiv='Potential error' then mederr = 'Potential error';

else if mederr='Intercepted medication error' or mederr_indiv='Intercepted medication error' then mederr = 'Intercepted medication error';

else if mederr='Medication with harm' or mederr_indiv="Medication with harm" then mederr='Medication with harm';

else if mederr='Medication without harm' or mederr_indiv="Medication without harm" then mederr='Medication without harm';

end;

if last.vcdoseid then do;

if mederr="OFF" then mederr="";

output;

end;

run;

%if "&outdsn." eq "%str()" %then %do;

proc sql undo_policy=none;

create table &inpsrc. as

select distinct a.*, b.mederr

from &inpsrc. as a left join medErr_aggregate as b on a.csc=b.csc and a.vcdoseid=b.vcdoseid and a.vcfamname=b.vcfamname

order by csc, vcdoseid, evsoc, evpt, evhlgt, evhlt;

quit;

%end;

%exit:

%mend MedErr;
